# Mobile health platform based on user-centered design to promote exercise for patients with peripheral artery disease

**DOI:** 10.1186/s12911-022-01945-z

**Published:** 2022-08-02

**Authors:** Mihui Kim, Yesol Kim, Mona Choi

**Affiliations:** 1 College of Nursing and Brain Korea 21 FOUR Project, Yonsei University, 50-1 Yonsei-ro, Seodaemun-gu, Seoul, 03722 Republic of Korea; 2 College of Nursing and Mo-Im Kim Nursing Research Institute, Yonsei University, 50-1 Yonsei-ro, Seodaemun-gu, Seoul, 03722 Republic of Korea

**Keywords:** Exercise, Health behavior, Mobile applications, Mobile health platform, Peripheral artery disease

## Abstract

**Background:**

Peripheral artery disease (PAD) is a cardiovascular disease that can be improved by risk factor modification. Mobile health (mHealth) intervention is an effective method of healthcare delivery to promote behavior changes. An mHealth platform can encourage consistent involvement of participants and healthcare providers for health promotion. This study aimed to develop an mHealth platform consisting of a smartphone application (app) synchronized with a wearable activity tracker and a web-based portal to support exercise intervention in patients with PAD.

**Methods:**

This study was conducted based on an iterative development process, including analysis, design, and implementation. In the analysis phase, a literature review and needs assessment through semi-structured interviews (*n* = 15) and a questionnaire-based survey (*n* = 138) were performed. The initial prototype design and contents were developed based on the users’ requirements. In the implementation phase, multidisciplinary experts (*n* = 4) evaluated the heuristics, following which the mHealth platform was revised. User evaluation of the usability was performed by nurses (*n* = 4) and patients with PAD (*n* = 3).

**Results:**

Through the development process, the functional requirements of the platform were represented through visual display, reminder, education, self-monitoring, goal setting, goal attainment, feedback, and recording. In-app videos of exercise and PAD management were produced to provide information and in-app automatic text messages were developed for user motivation. The final version of the platform was rated 67.86 out of 100, which indicated “good” usability.

**Conclusions:**

The mHealth platform was designed and developed for patients with PAD and their healthcare providers. This platform can be used to educate and promote individualized exercise among patients with PAD.

## Background

Peripheral artery disease (PAD) is a cardiovascular disease, characterized by walking difficulties due to intermittent claudication defined by leg muscle pains, fatigue, or cramping induced by exercise or ambulation, and its prevalence rising with age [1, 2]. Notably, declined functional status and PAD-related symptoms are associated with poorer health-related quality of life [2, 3]. PAD prognosis can be improved by risk factor modification through revascularization, medication, and exercise therapy [4]. Exercise therapy involving moderate or high-intensity walking is an important element in improving the functional status and quality of life in patients with PAD [2].

Mobile health (mHealth) technology uses wireless devices and sensors to facilitate exercise adherence [5, 6] and includes a smartphone application (app) or wearable activity trackers. A combination of effective individual feedback and mHealth technology has been demonstrated as an effective strategy for promoting health behavior change [7, 8] and improving health outcomes [5, 9, 10] in several populations, including patients with acute or chronic conditions. Based on the evidence, it is assumed that the positive health-related benefits observed in these populations may extend to patients with PAD.

Smart devices such as smartphones, tablet PCs, and wearable devices have been extensively used for mHealth interventions [11]. In patients with PAD, the devices used for mHealth-based interventions are mainly wearable activity trackers [7, 12–16], and additional smartphone apps have been used in recent studies [17–19]. The features of these smartphone apps include monitoring the patient’s activity and pain levels, maintenance of a health diary, and health coaching. Information and motivation are important to promote behavior change [20], which can be provided using smartphone app features that cater to exercise intervention [21]. Smartphone apps for patients with PAD have provided motivation through activity monitoring and coaching to encourage exercise; however, there have been limitations in providing educational information, such as disease progression, risk factor management for PAD deterioration, and suitable exercise methods for PAD. Approximately 95% of Korean adults own smartphones, and 85% of those aged 60 or older have smartphones with access to the internet and app [22]; therefore, it may be highly feasible to use a smartphone app for exercise-based intervention.

According to the systematic review, exercise programs incorporating behavior change strategies, such as feedback, guidance, and monitoring based on set goals and using wearable activity trackers in patients with PAD, improve walking ability and exercise adherence rate [12] (degree of performance according to set goals). mHealth interventions that promote physical activity can enable goal setting, self-monitoring, feedback, rewards, sharing, and social comparison [7, 23]. Exercise interventions in patients with PAD along with wearable activity trackers for precise activity measurement and smartphone apps for intensive interaction, may provide richer content through various delivery modes. Therefore, an mHealth platform, consisting of a smartphone app synchronized with a wearable activity tracker, and a web-based portal, may be suitable for supporting and managing exercise in patients with PAD.

The mHealth platform allows patients and healthcare providers to monitor and manage data in real-time [24–26]. In this study, the mHealth platform was a Home-Based Behavior Intervention with Technology for Peripheral Artery Disease (HOBBIT-PAD) platform to promote exercise for the group of patients. This study was conducted based on the information-motivational-behavioral skills (IMB) model [20], which can explore and reflect behavior change mechanisms. This model explains that adherence-related information and motivation improve behavior skills and promotes behavior change [20]. It is essential to involve users early in the app development process to provide information and motivation suitable for user needs [27, 28].

This study aimed to develop the HOBBIT-PAD platform based on a user-centered design to support exercise intervention in patients with PAD based on the IMB model. The specific objectives for the development of the platform were as follows: (1) to identify the requirements by analyzing data through a literature review and undertaking an assessment of the users’ needs; (2) to design and develop the platform based on the functional requirements that were identified; and (3) to evaluate the developed platform by multidisciplinary experts, healthcare providers, and users.

## Methods

**Fig. 1:**
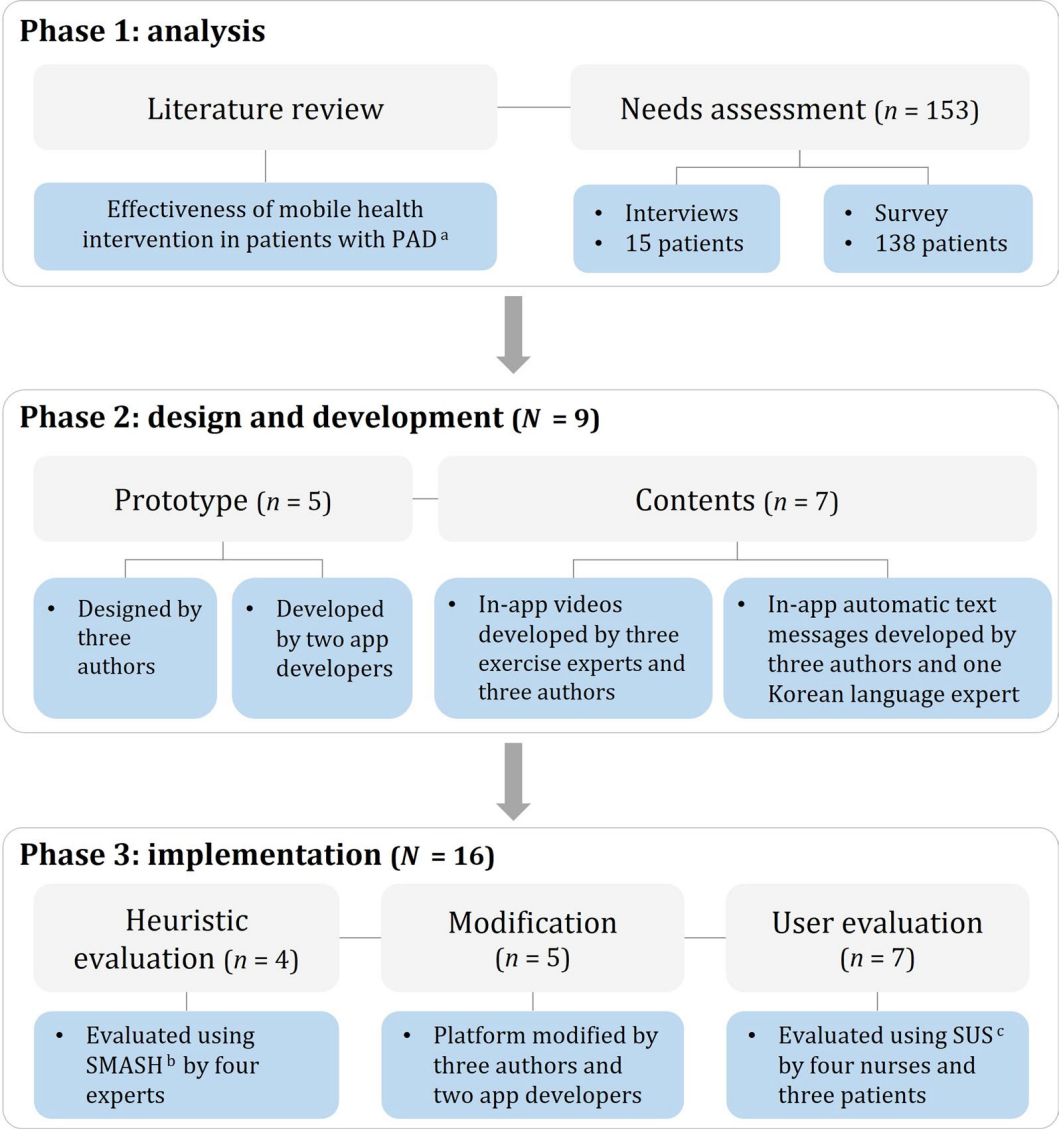
Description of the mHealth app development process. ^a^ Peripheral artery disease, ^b^ SMArtphone’s uSability Heuristics, ^c^ System usability scale

The software development process was based on iterative development using a user-centered design approach to promote behavior change, which involves the following three phases: analysis, design, and implementation [28, 29] (Fig. 1).

This iterative development process is continued until the software reaches a prespecified satisfactory level indicated in the system requirements specification, and teamwork is emphasized to develop, test, modify, and retest periodically during the process for high-quality software [29]. In the user-centered design approach, it is important to accurately assess the end-users’ needs and develop systems that focus on meeting their requirements [27, 28]. Therefore, this study included patients with PAD in the development process (Phases 1 and 3), accurately assessed their requirements and priorities with regard to smartphone apps for behavior change, and evaluated the usability of the smartphone app.

In total, 173 participants were involved in the development process: Phase 1 involved 153 patients; Phase 2 involved nine participants (three authors, two app developers, three exercise experts, and one Korean language expert); and Phase 3 involved 16 participants. The authors of this study participated in all three phases and played the roles in conducting the study, designing the prototype, producing videos, and developing automatic text messages.

### Phase 1: analysis

#### Literature review

The authors had previously conducted a systematic review and meta-analysis to assess the effectiveness of mHealth interventions in patients with PAD. The details of these methods have been published elsewhere [12]. The results of the review identified the important and commonly used features and additional necessary functions in mHealth intervention.

#### Needs assessment

End-user involvement is critical in smartphone app development [30]. To assess the practical needs for the HOBBIT-PAD platform, a semi-structured interview and a questionnaire-based survey were conducted on patients with PAD. The inclusion criteria were aged above 19 years old and diagnosed with PAD. Patients who met the eligibility criteria for the study were recruited with the help of medical staff at the cardiovascular outpatient clinic of a tertiary hospital in Seoul, Korea. Outpatient nurses informed authors when they identified visiting patients who met the eligibility criteria, and authors explained the purpose and process of this study and obtained written consent from patients who voluntarily agreed to participate in this study.

The semi-structured interviews were conducted on 15 participants using purposeful sampling from February to July 2020. The interviews were conducted in an independent space within the hospital, and the data were collected through voice record and field notes. The main interview questions were “What do you think is the most important part of exercising by yourself?”, “What is the greatest difficulty in exercising by yourself?”, and “What would be the most important functions of a smartphone app developed for patients with PAD?” The recorded interviews were transcribed verbatim and analyzed using content analysis.

For the questionnaire-based survey, 138 patients were recruited from June to October 2020. The seven questions derived from the interviews assessed the level of information needed related to exercise, diet, smoking, alcohol, and information resources related to PAD, essential functions of a smartphone app to be developed, and intention to use a developed smartphone app for PAD. The data were analyzed and are presented as mean and standard deviation or frequency and percentage.

Based on findings from interviews and questionnaire-based surveys, the requirements of the HOBBIT-PAD platform were identified. These requirements, including patient expectations, consisted of the specifications for the design and content of the platform.

### Phase 2: design and development

#### Prototype

The initial prototype was constructed based on the users’ requirements for a smartphone app for behavior change based on user-centered design, and the app developers undertook the HOBBIT-PAD platform development based on the prototype design. An initial version of the platform was built and tested to ensure it was bug-free by the authors. A smartphone app was developed on the Android operating system (version 4.4 or higher).

#### Contents

In-app videos related to exercise and PAD were created to provide exercise-related information to patients. The exercise video files were made by exercise experts, and videos related to disease management were made by the authors according to results from the patients’ needs assessment.

A text message library based on the behavior change wheel (BCW) framework was developed to motivate patients to exercise [31]. The BCW allows the selection of interventions according to the analysis of target behaviors that need to be changed. The target behavior of this study was set to induce a change from sedentary to walking behavior, and the barriers to performing this change were identified as leg pain, poor physical condition, forgetting to exercise, unwillingness to exercise, bad weather, and lack of time. As a strategy to overcome these barriers, the authors linked them to seven interventions (training, enablement, education, persuasion, incentivization, coercion, and environmental restructuring) that can be applied to the development of in-app automatic text messages. In-app text messages were automatically delivered via app notifications to encourage exercising according to the individual exercise barriers ascertained from the daily ecological momentary assessment (EMA) survey, as described in a previous study [32].

### Phase 3: implementation

#### Heuristic evaluation

The heuristic evaluation of the smartphone app was performed by four experts with majors in computer sciences and nursing informatics and with experience in mobile app development and design. They evaluated the smartphone app based on 12 heuristics items according to the Korean version of the SMArtphone’s uSability Heuristics (SMASH) [33]. Each heuristic item was rated on a Likert five-point scale ranging from 0, “No problem” to 4, “Usability catastrophe” [34]. Since the smartphone app was developed on an Android operating system, app developers built the web version to test it in the same environment as the smartphone app. The experts evaluated the app individually after using the web version.

#### Modification

Among the 12 heuristic items, items that received a rating of 3, “important to fix, so should be given high priority”, and those receiving 4, “imperative to fix this before the product can be released”, were modified. The feedback on the evaluation was incorporated into the smartphone app’s functions after discussions between the research team.

#### User evaluation

User evaluation was performed by four nurses and three patients with PAD. Nurses with more than five years of experience in caring for PAD and patients with PAD who used an Android smartphone were recruited. Nurses were provided with a web version of the app to evaluate, while patients were provided with the smartphone app with a wearable activity tracker and asked to use them daily for seven days.

The user evaluation was conducted using the system usability scale (SUS) and additional open-ended questions on the app. SUS is a valid and reliable questionnaire [35] and this study used the Korean version of the SUS [36]. In the SUS, odd-numbered items are positive statements, and even-numbered items are negative statements so as avoid response biases. Each item is rated on a five-point Likert scale ranging from 1 (strongly disagree) to 5 (strongly agree). “Strongly disagree” with a negatively worded item is equivalent to “strongly agree” with a positively worded item. The total SUS score is calculated by multiplying the weight and ranges from 0 to 100. The higher the SUS score, the higher the perceived usability. Based on the SUS score, usability was classified as “best imaginable, excellent, good, OK, poor, or worst imaginable” [37]. Through open-ended questions, additional opinions on the app were collected from users. The following three questions were used: “What were the good points about using this app?”, “What was the inconvenience of using this app?”, and “Do you have any improvements or suggestions for this app?”.

## Results

### Phase 1: analysis

#### Literature review

In our prior work [12], seven randomized controlled trials on mHealth intervention for patients with PAD were reviewed, and all included studies used wearable activity trackers to measure daily steps. The intervention functions of included studies consisted of recording and displaying the number of steps through a wearable activity tracker, providing feedback regarding goal attainment, and issuing reminders to remain active. Real-time intervention, an advantage of mHealth, was not applied to monitoring and feedback on individually set goals and symptoms. A total of six studies included in the meta-analysis showed that mHealth-based exercise interventions at home improved pain-free and maximal walking times and the 6-minute walking test distance compared to the usual care group. Additionally, it was reported that mHealth technology would help maintain adherence to exercise goals in home-based interventions.

Based on findings from the literature review, the HOBBIT-PAD platform consisted of a smartphone app paired with a wearable activity tracker and a web-based portal to enable real-time feedback, monitoring, and recording of patients’ daily steps, symptoms, and goal attainments [12]. The recorded data were used to provide daily reminders and feedback to maintain exercise based on whether the individually set goal was achieved.

#### Needs assessment

A total of 15 patients aged 32–82 years (11 males and 4 females; median age: 74 years) participated in semi-structured interviews. A key finding was that all participants were aware of the importance of exercise and exercise programs for disease management.

*“There are educations for diabetic patients on how to manage diet and other things, but there is no program for patients with peripheral artery disease who underwent revascularization. (Participant 6)*”

“*At first, I liked to stay at home without walking. I think it’s better to walk now. That’s the best and the easiest. (Participant 11)*”

The participants answered that the most important part of the exercising by oneself was lifestyle modification, and the most difficult part was exercising alone without supervision and reverting to their previous lifestyles.

“*I couldn’t exercise a lot… I want someone to lead me. (Participant 5)*”

“*Because there is nothing wrong with my body, I have gradually forgotten about exercising. (Participant 2)*”

Participants’ expectations of the smartphone app included availability of tailored exercise prescriptions based on their condition, display of daily activities, provision of educational information related to PAD, regular and timely exercise reminders, provision of emotional support, and use of easy-to-understand terms.

A total of 138 patients participated in the survey, with an average age of 69.04 (± 10.94) years; 91.3% were male. Patients expected to receive information on exercise (73.9%) and diet (64.5%). Most patients (84.8%) obtained PAD-related information from doctors or nurses. Participants expected the smartphone app to include details of PAD management (78.3%), exercise (75.4%), diet (60.1%), and health status records (35.5%). Approximately 85% of the patients were willing to use a smartphone app if it were developed.

Through the interviews and survey, the functional requirements of the HOBBIT-PAD platform based on the IMB model were summarized (Table 1). In addition, the functions of the platform were mapped to 12 techniques among the 26 behavior change techniques [38] to provide effective intervention.

**Table 1:** Functional requirements of the platform

Category	Function	Content	Behavior change technique	mHealth platform		
				App	Wearable tracker	Web-based portal
Information	Visual display	Personal graph of exercise	Prompt self-monitoring of behavior	◯	◯	◯
	Reminder	Exercise time	Prompt practice	◯		◯
	Education	Exercise, disease management	Provide information about behavior-health link and others’ approval and consequence, Model or demonstrate the behavior	◯		◯
Motivation	Self-monitoring	Daily/weekly exercise	Prompt self-monitoring of behavior	◯	◯	◯
	Goal setting	Assigning tailored step count goal	Prompt specific goal setting, Set graded tasks	◯		◯
	Goal attainment	Reward depending on achievement	Provide contingent rewards	◯		◯
	Feedback	Automatic text messages	Prompt intention formation, Provide feedback on performance	◯		◯
	Recording	Activities, pain level	Prompt self-monitoring of behavior, Prompt barrier identification	◯		◯

### Phase 2: design and development

#### Prototype

**Fig. 2:**
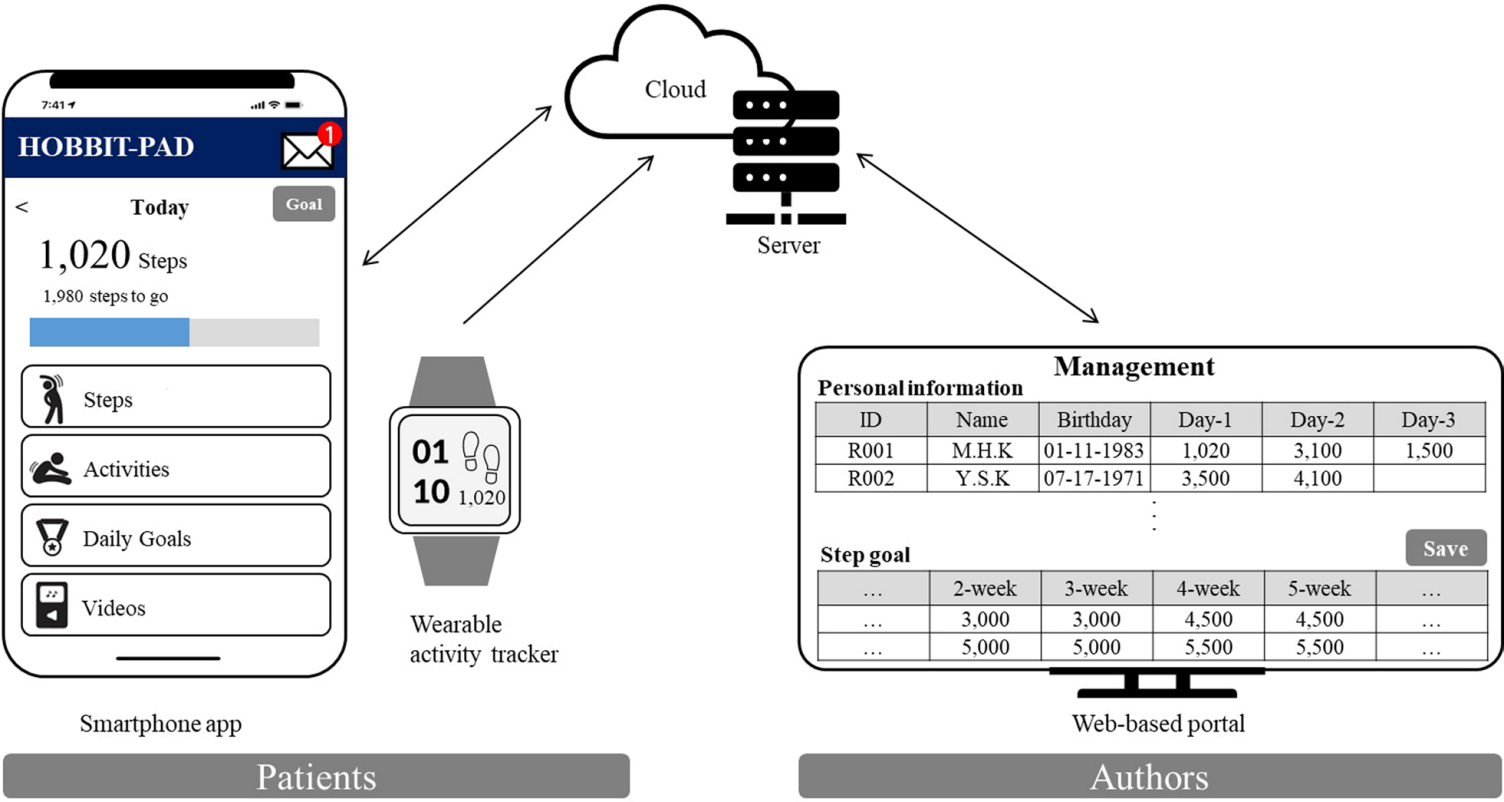
Prototype of the HOBBIT-PAD platform

The authors designed a comprehensive HOBBIT-PAD platform based on user-centered design to provide information and motivation through functional requirements (Fig. 2). The smartphone app was synchronized with a wearable activity tracker, the Fitbit Charge 4 (Fitbit Inc., San Francisco, CA, USA). Its menu included the following: displaying steps, activities, daily goals, videos, goal, and message. The web-based portal used by the authors included the following features: participant management, monitoring (exercise and survey), and goal settings for each patient.

The menu and functions of the apps used by patients are shown in Fig. 2. The “Steps” menu displayed the number of daily and weekly steps paired with the Fitbit charge 4 worn by patients; the “Activities” menu recorded daily exercise. In the “Daily Goals” menu, one could check the attained daily target steps set in the “Goal” menu. In the “Videos” menu, videos on exercise and disease management were included. In the “Message” menu, the patient could record whether to exercise, exercise barrier, and lower extremity pain level through daily app push and receive an automatic text message to encourage exercise according to the selected exercise barrier.

The main menus of the web-based portal used by the authors were Monitoring of Exercise and Survey. The “Monitoring of Exercise” menu displayed the individual goals and steps paired with the Fitbit charge 4 for all patients. In addition, the “Monitoring of Survey” menu was also monitored by the authors for individual daily survey results that patients recorded in their app’s “Message” menu.

#### Contents

A total of five in-app exercise videos were produced by exercise experts with a playback time of approximately 5 to 14 min. These videos were used as a visual demonstration with music and explained how to perform each exercise: a warm-up and cool-down exercise, walking, and muscle-strengthening activities. All videos were reviewed and checked by the authors.

A total of four in-app videos related to PAD management were produced by the authors based on a literature review focused on easily understandable content. These videos covered disease prognosis, lifestyle management (hypertension, diet, and smoking), and the importance of exercise.

A text message library with 120 messages and EMA based on the BCW framework was developed in a previous study [32] and used for in-app automatic text messaging to encourage participants to exercise.

### Phase 3: implementation

#### Heuristic evaluation

Four experts (one male and three females; aged 31–42 years) conducted a heuristic evaluation using the Korean version of SMASH. They were one nursing professor with a nursing informatics major, one computer scientist, and two with experience in mobile app development and design. Among the 12 heuristic items, six items scored 3 or 4 points from experts and thus required modification (Table 2). The other items were scored from 0 to 2. In all, 40 feedbacks were received from experts on features of the smartphone app that needed improvement and specifically described in the modification part.

**Table 2:** Heuristic evaluation of smartphone application

The Korean version of SMASH ^a^	Expert 1	Expert 2	Expert 3	Expert 4
1. Visibility of system status	1	1	0	3
2. Match between system and the real world	0	2	0	0
3. User control and freedom	2	2	0	2
4. Consistency and standards	1	0	1	2
5. Error prevention	2	0	0	4
6. Minimize the user’s memory load	0	0	0	2
7. Customization and shortcuts	0	1	0	1
8. Efficiency of use and performance	1	1	0	3
9. Esthetic and minimalist design	0	1	1	4
10. Help users recognize diagnose, and recover from errors	0	2	0	0
11. Help and documentation	3	2	0	4
12. Physical interaction and ergonomics	0	0	0	4

^a^ SMArtphone’s uSability Heuristics

0 = No problem: I don’t agree that this is a usability problem at all, 1 = Cosmetic problem only: does not need to be fixed unless extra time is available on the project, 2 = Minor problem: fixing this should be given low priority, 3 = Major problem: important to fix, so should be given high priority, 4 = Usability catastrophe: imperative to fix this before product can be released

#### Modification

The results of the heuristic evaluation were divided into errors and modifications. Experts who majored in design mainly suggested extensively revising the design aspects of the app, such as graphic presentation and color. Through discussion, the authors prioritized items that required modifications. Finally, all errors were corrected, and the selected modifications were made (Table 3).

**Table 3:** Modification of smartphone application according to a heuristic evaluation

Menu	Contents	Before	After
Main screen	Modifying the graph and text due to overlap with the “steps” menu, - Deleting of the “Exercise program” banner at the bottom of all screens		
Activities	Combining activities in one button as a graphic for simplicity		
Daily goals	Adding buttons to jump to another month on the calendar		
Automatic text-message	Changing the title to the “Research team” who sent the message and the color of the messages		

**Fig. 3:**
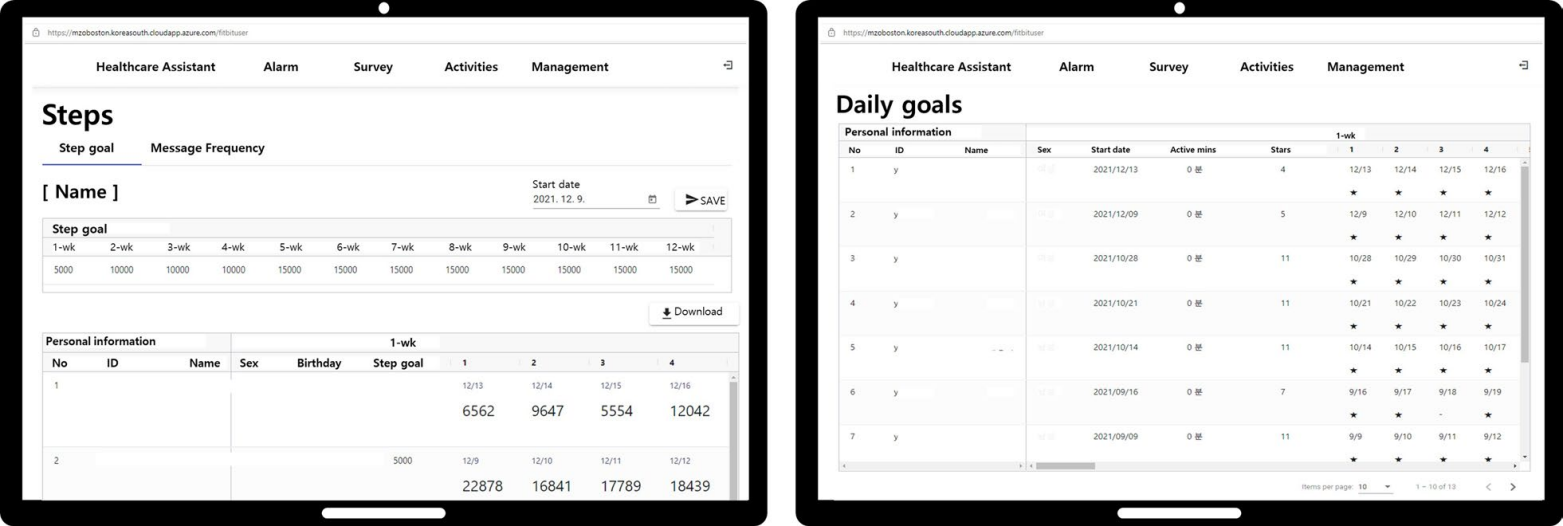
Screenshot of the web-based portal

**Fig. 4:**
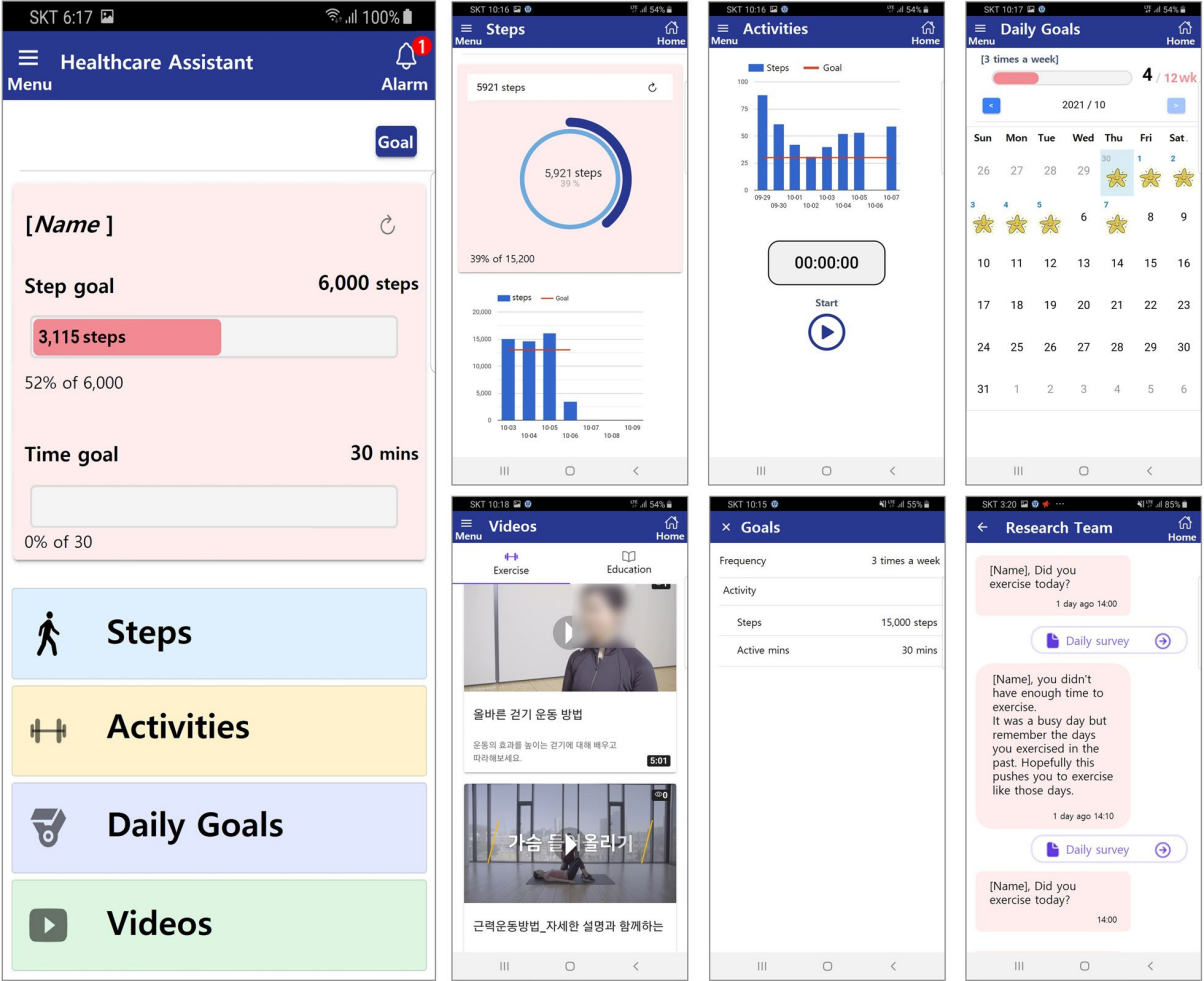
Screenshots of the smartphone app

The final version of the web-based portal was connected through a URL (Fig. 3), and the smartphone app (Fig. 4) could be installed by scanning a QR code or via a URL.

#### User evaluation

Four nurses (all females; aged 30–32 years) and three patients with PAD (all males; aged 62–69 years) participated in the user evaluation. The mean SUS total score for nurses was 75.63, indicating “excellent”. The mean SUS total score for patients with PAD was 57.50, indicating “good”. The overall SUS total score ranged from 32.50 to 82.50 with an average of 67.86, which indicated “good” (Table 4).

**Table 4:** System usability scale mean scores

Items ^a^	Nurses (*n* = 4)	Patients (*n* = 3)	Total (*n* = 7)
1. I think that I would like to use this system frequently	3.75	3.67	3.71
2. I found the system unnecessarily complex ^b^	1.75	2.33	2.00
3. I thought the system was easy to use	2.75	3.00	2.86
4. I think that I would need the support of a technical person to be able to use this system ^b^	2.00	2.67	2.29
5. I found the various functions in this system were well integrated	4.00	4.00	4.00
6. I thought there was too much inconsistency in this system ^b^	1.25	2.67	1.86
7. I would imagine that most people would learn to use this system very quickly	4.50	3.00	3.86
8. I found the system very cumbersome to use ^b^	1.25	3.00	2.00
9. I felt very confident using the system	4.25	3.00	3.71
10. I needed to learn a lot of things before I could get going with this system ^b^	2.75	3.00	2.86
Total (Range: 0 ~ 100)	75.63	57.50	67.86

^a^ Each item is rated from 1 (strongly disagree) to 5 (strongly agree)

^b^ Negative statement

After using the developed app, the users’ opinions were collected through open-ended questions. First, the responses to the question on the good points of the app were as follows: uncomplicated and simple design, easy functions, ease of use, and encouragement for exercise through goal visualization, messages, and videos. Second, the responses to the question on the inconvenience of the app were as follows: need time to understand each item, simple and monotonous function and design, and the possibility of a decrease in the interest over time. Last, the opinions on improvement or suggestions for the app were as follows: upload additional videos regularly, add a description and guide for each item, add functions to motivate when logging in, provide positive feedback to the responses about doing exercise in the survey, and insert color or intuitive pictures to the background in the survey.

## Discussion

This study performed the iterative development process using user-centered design approach to develop the HOBBIT-PAD platform to support exercise behavior in patients with PAD, based on the IMB model [20, 28, 29]. The HOBBIT-PAD platform consisted of a smartphone app synchronized with a wearable activity tracker and a web-based portal to provide intensive interaction coupled with various functions and contents. The contents of this platform focused on providing motivation and information to improve behavior skills and promote behavior change based on the theoretical foundations.

The HOBBIT-PAD platform was developed with adequate consideration of the user’s needs and preferences through a user-centered design approach. The mixed methods were used to accurately assess user requirements for a smartphone app. The user requirements were designed with respect to each smartphone app screen, keeping in mind the ease of use for patients with PAD, the prevalence of which is rising with age. The smartphone app had minimum essential functions and attempted to increase the intuition of use through a simple design that could contribute to its willingness to use [39]. In addition, these essential functions were provided along with the behavior change techniques [23, 38] based on the users’ requirements.

Linking the theory and intervention content improves the efficacy of the intervention by identifying the constructs to be targeted and the mechanism of behavior change [38, 40, 41]. Therefore, the platform incorporated 12 behavior change techniques to provide effective intervention. Furthermore, to optimize the effectiveness of the mHealth platform, its contents were consisted accordingly to provide information and motivation for behavior change on the basis of the IMB model. The HOBBIT-PAD platform consisted of information delivered by visual display, reminders (exercise time), education (in-app videos), and motivation given by self-monitoring, goal settings, goal attainment, feedback, and recording.

As a result of the heuristic evaluation of a smartphone app by experts, 6 out of 12 items needed modifications. The priorities and feasibility of these items were also considered and modified. The modified smartphone app has enabled system improvements and a convenient and easy interface for older adults. As a result, the finding of usability evaluations of the modified smartphone app was rated to have good acceptability to users.

Through the HOBBIT-PAD platform, patients with PAD accomplished self-monitoring and received real-time feedback, and the authors could accurately monitor patients’ activity and pain levels. Therefore, it may replace a supervised exercise program by providing assistance and support to patients with PAD transitioning from in-person, supervised exercise programs to an independent home-based exercise program. In other studies, mHealth platforms have been developed for exercise-based cardiac rehabilitation [26], Parkinson disease management [25], and HIV care [24] that can effectively support patients or caregivers and healthcare providers with consistent involvement. The HOBBIT-PAD platform can be an integrated system that monitors and stores data in real time, and offers feedback with minimum effort. Therefore, by providing exercise intervention to patients with PAD using the platform, it can be expected that the behavior skills and exercise adherence rate may be improved through information and motivation, ultimately inducing a change in the targeted behavior.

This study has several limitations. First, the smartphone app was only available for the Android operating system. As approximately 80% of people in Korea use Android smartphones, and its use increases with age [22], the authors chose to develop an Android smartphone app that could be used by more patients, owing to limited resources. Secondly, in the third phase, we evaluated the smartphone app only. Although the web-based portal was periodically tested and retested by authors focusing on technical errors during the development process, the portal needs to be evaluated by experts or healthcare providers to determine the usability. Finally, the smartphone app was developed to synchronize with Fitbit Charge 4. Therefore, an additional development process is required when synchronized with other commercial wearable activity trackers.

## Conclusions

The HOBBIT-PAD platform was designed and developed to provide a system for patients with PAD and their healthcare providers. This platform may promote behavior changes in patients with PAD in their everyday lives and easily provide patients’ status to healthcare providers. The user-centered and safe smartphone app synchronized with a wearable activity tracker may be possible to promote exercise in patients with PAD. The HOBBIT-PAD platform with real-time interaction can be applied to patients with PAD, and further evaluation of its effectiveness and acceptability is expected.

## Data Availability

The data generated during the current study are not publicly available due to the need to maintain the anonymity of participants and the confidentiality of the data. However, the data are available from the corresponding author on reasonable request.

## Abbreviations

App: Application
BCW: Behavior change wheel
EMA: Ecological momentary assessment
IMB: Information-motivational-behavioral skills
mHealth: Mobile health
PAD: Peripheral artery disease
SMASH: SMArtphone’s uSability Heuristics
SUS: System usability system
